# Analysis of Philip Morris International’s ‘aspirational’ target for its 2025 cigarette shipments

**DOI:** 10.1136/tc-2023-058511

**Published:** 2024-05-23

**Authors:** John Mehegan, Allen Gallagher, Sherif Elmitwalli, Richard Edwards, Anna Gilmore

**Affiliations:** 1 Department for Health, University of Bath, Bath, UK; 2 Public Health, University of Otago, Wellington, New Zealand

**Keywords:** Economics, Global health, Tobacco industry, Surveillance and monitoring

## Abstract

**Background:**

Philip Morris International (PMI) claims to be transforming and has committed to a ‘smoke-free’ future. In 2020, it announced an ‘aspirational’ target for reduced cigarette shipments by 2025.

**Methods:**

PMI cigarette shipment data are taken from PMI quarterly financial reports 2008–2023. Trends in these data before and after the 2020 announcement are analysed using linear regression, and auto regressive integrated moving average and error, trend, seasonal time-series models to assess if PMI’s 2025 target would be met on pre-existing trends, and if the trend changed after the announcement. These trends are also compared with the global retail market for cigarettes, using sales data from Euromonitor.

**Results:**

Findings were consistent across all three models. PMI’s shipment target of 550 billion cigarette sticks by 2025 would readily have been met given pre-existing shipment trends. Following the 2020 announcement, the decline in PMI cigarette shipments stalled markedly with a statistically significant change in trend (p<0.001). The current and projected trend to 2025 is consistent with no further decline in cigarette volumes, meaning PMI is unlikely to hit its target. This mirrors a global pattern in which declines in cigarette sales have stalled since 2020.

**Conclusions:**

PMI’s 2025 target was not ‘aspirational’ but highly conservative—it would have been met based on pre-existing trends in declining cigarette shipments. Yet PMI will nonetheless fail to meet that target providing evidence it is not transforming. Stalling of the decline of PMI and global cigarette sales raises significant concerns about progress in global tobacco control.

**WHAT IS ALREADY KNOWN ON THIS TOPIC**

- One of the world’s largest cigarette companies, Philip Morris International (PMI), has since 2016 very publicly claimed to be transforming and has committed to achieving what it calls a ‘smoke-free’ future.
- In 2020, PMI set what it labelled an ‘aspirational’ target for reducing its own cigarette shipments. If PMI’s claims of ‘transformation’ are to be believed, this target and the trends in its cigarette sales need to be robustly evaluated.

**WHAT THIS STUDY ADDS**

- A detailed examination of PMI’s shipment volumes and sales showed that its 2025 target for reduced cigarette shipments was very conservative and would be met on pre-2020 trends.
- Moreover, post-2020 trends show that PMI cigarette shipments are barely declining, and the company is not on track to meet its own conservative 2025 target.

**HOW THIS STUDY MIGHT AFFECT RESEARCH, PRACTICE OR POLICY**

- This study provides a robust approach to analysing cigarette sales and shipment trends, an important aspect of understanding and monitoring the activities of tobacco companies.
- The observed stalling in cigarette shipments and sales post-2020 is worrying from a public health standpoint and emphasises the need for continued vigilance from policy-makers and tobacco control advocates.

## Introduction

 In 2016, Philip Morris International (PMI), one of the world’s largest transnational tobacco companies, claimed to be ‘transforming’ by committing to a ‘smoke-free’ future in which it would minimise sales of combustible products and focus on what it describes as its ‘smoke-free’ products, notably its heated tobacco product (HTP) ‘IQOS’, which launched in 2014.^1^ The extent to which products such are IQOS are accurately represented by terminology such as ‘smoke-free’ has been called into question in recent literature.^2^ However, the focus of this paper is not on assessing this, or indeed looking at the trajectory of PMI’s HTP business, but on determining whether PMI’s cigarette shipment and sales activities are consistent with a change in business direction and whether its aspirational claims about reducing these shipments and sales are in line with a proposed definition and essential criterion for tobacco industry transformation: the rapid elimination of the production, distribution, marketing and sales of conventional smoked tobacco products.^3^

The aspirational claims in question stem from multiple PMI publications dating back to 2020. In its 2019 Integrated Report, released 30 June 2020, PMI set ‘aspirational targets’ for the sale of its products: that by 2025, shipment volume of its cigarettes would decrease by 22% to less than 550 billion units, from 707 billion units in 2019 and that, simultaneously, shipment volume of its ‘smoke-free’ products would increase more than fourfold to over 250 billion units, from 60 billion units in 2019.^4^ PMI restated these targets in its 2022 Integrated Report, released 5 April 2023, further adding that it aimed for more than half of its net revenues to come from its ‘smoke-free’ products by 2025 while ‘continuing to reduce’ its cigarette shipment volumes.^5^ Such claims coincided with an intense period of industry lobbying and self-promotion, with PMI in particular claiming to be the solution to the smoking epidemic it had helped create.^6^

Despite its claimed commitment to a smoke-free future, PMI has also stated that it aims to maintain its leadership position in the combustible products market,^7^ with the justification that its transformation to ‘smoke-free’ products requires funding from its cigarette business.^8^ In line with this, evidence suggests that PMI’s claims that it has ‘transformed’ are questionable,^3 9^ with the company having since continued to launch new cigarette brands, buy up new cigarette companies and oppose effective tobacco control policies.^6^

This paper explores whether PMI’s target for reducing cigarette shipments can be viewed as an aspirational shift in the company’s business model (away from reliance on combustible products) or simply reflects pre-existing trends in cigarette sales and shipments when the target was announced. Relatedly, it also examines whether the trend in cigarette shipments and sales has changed since the announcement of the 2025 target, in line with PMI’s claim of adopting a transformation agenda.

## Methods

### Data sources

Data on PMI cigarette shipment volumes are taken from PMI’s quarterly financial reports for the period quarter 1 (January–March) 2008 to quarter 3 (July–September) 2023.^10^ Quarterly data are chosen as they are the most complete and granular level of shipment data available and allow the application of standard time-series analysis techniques to investigate trends and forecast future volume levels.

Data on cigarette sales, across companies and regions, were extracted from the market research company Euromonitor’s online database.^11^ Euromonitor provides data and analysis on multiple industries, including tobacco, collecting data through a variety of sources, including surveys, industry reports and government data and is a common source for market data globally.^11^ It provides full-year annual sales and revenue data for the tobacco sector, with the data updated annually in late spring. Data from Euromonitor were extracted in July 2023 for the period 2008–2022 (2008 being the year PMI split from its former parent company Altria and 2022 being the latest full year for which data are available). Note that at the data extract date (July 2023) Euromonitor provided overall global and region-specific cigarette sales data from 2008 onwards, but company-specific sales data only from 2013 onwards.

### Data analysis

This paper primarily investigates PMI’s 2025 cigarette shipment target against its own reported shipment volumes. However, it also compares PMI shipment data to Euromonitor sales data, for overall global cigarette sales (excluding China), with and without including PMI sales data. To this end, first PMI’s reported annual cigarette shipments are compared with PMI annual cigarette sales data from Euromonitor for the study period where Euromonitor data are available (2008–2022), with trends in sales and shipment volumes compared via calculation of percentage changes, cumulative totals and simple linear fits.

Identifying 2012 as an inflection point where PMI cigarette shipments began to fall, an analysis of the trends in PMI quarterly cigarette shipments is undertaken for the periods before and after PMI’s 2019 Integrated Report announcement, respectively, up to the end of 2019 from Q1 2012 to Q4 2019 (labelled ‘pre-2020’ hereafter), and from the start of 2020, Q1 2020 to Q3 2023 (labelled ‘post-2020’ hereafter).

Three approaches to analysing these trends are used: (1) a simple linear regression of the cigarette shipment volumes against quarterly period, (2) an auto regressive integrated moving average (ARIMA) model and (3) an error, trend, seasonal (ETS) state space model. ARIMA and ETS are the two most widely used approaches to modelling time-series data, ARIMA primarily using autocorrelations in the data to model the time series, with ETS focusing on modelling the trend and seasonality in the data^12^ (see box 1). These three different approaches are chosen to check if modelled trends and forecasts are consistent across different analysis methodologies, thus adding to the robustness of the findings presented. R V.4.2.1 was used for the statistical analysis, with the core stats package function lm used for linear regression throughout, and the forecast package (V.8.21.1) functions auto.arima and ets used for ARIMA modelling and ETS modelling, respectively.^12^

Box 1 **ARIMA and ETS time-series models**

- In ARIMA models three variables p, d and q need to be chosen to find the ARIMA model that best fits the data: p is the number of previous time points (lag) included in the autocorrelation, d is the order of differencing between measurements that is used and m is the window size of any moving average terms used in the autocorrelations. By convention the labelling (p,d,q) is used to denote the model. ARIMA models applied to seasonal time-series data, like the quarterly shipment data, add further parameters P, D, Q which are the equivalent to p, d, q, respectively, but applied separately to the seasonal components of the data. Seasonal ARIMA models are labelled as (p,d,q) (P,D,Q)_m_, where m is the seasonality of the data (m=4 for quarterly data as in this study). The auto.arima function in R used in this study automatically determines the parameter set that best fits the data, choosing the model that minimises Akaike’s information criterion (AIC).^12 27^
- ETS models use underlying state equations including trend, seasonality and error to fit the time-series data. The choice in these models is between equations that include each of these terms multiplicatively, additively or not at all. The ets function in R also automatically determines these model choices to find the model that best fits the data, again choosing the model that minimises AIC.^12 27^
  ARIMA, auto regressive integrated moving average; ETS, error, trend, seasonal.

ARIMA and ETS models as applied to quarterly data can be used to forecast the point value of the series in each quarter into the future from the last fitted time point. In this study, the forecasts for individual quarters in each year are aggregated to provide yearly totals and averages, with these forecast yearly/quarterly averages reported and plotted in the results presented below.

Forecasts of future shipment volumes up to 2026 are determined using each of the three approaches. These are used to assess PMI’s 2025 cigarette shipment target, first against the pre-2020 trends, and subsequently to compare the pre-2020 and post-2020 trends to see if there is any change after the 2019 Integrated Report announcement.

Finally, to put the results in context, these trends in PMI cigarette shipment volumes are compared with trends in cigarette sales in the wider market, using total sales and sales of peer transnational tobacco companies at both a global and regional level. Compound annual growth rates are used to compare changes in these data over the time periods of interest.

## Results

**Figure 1:**
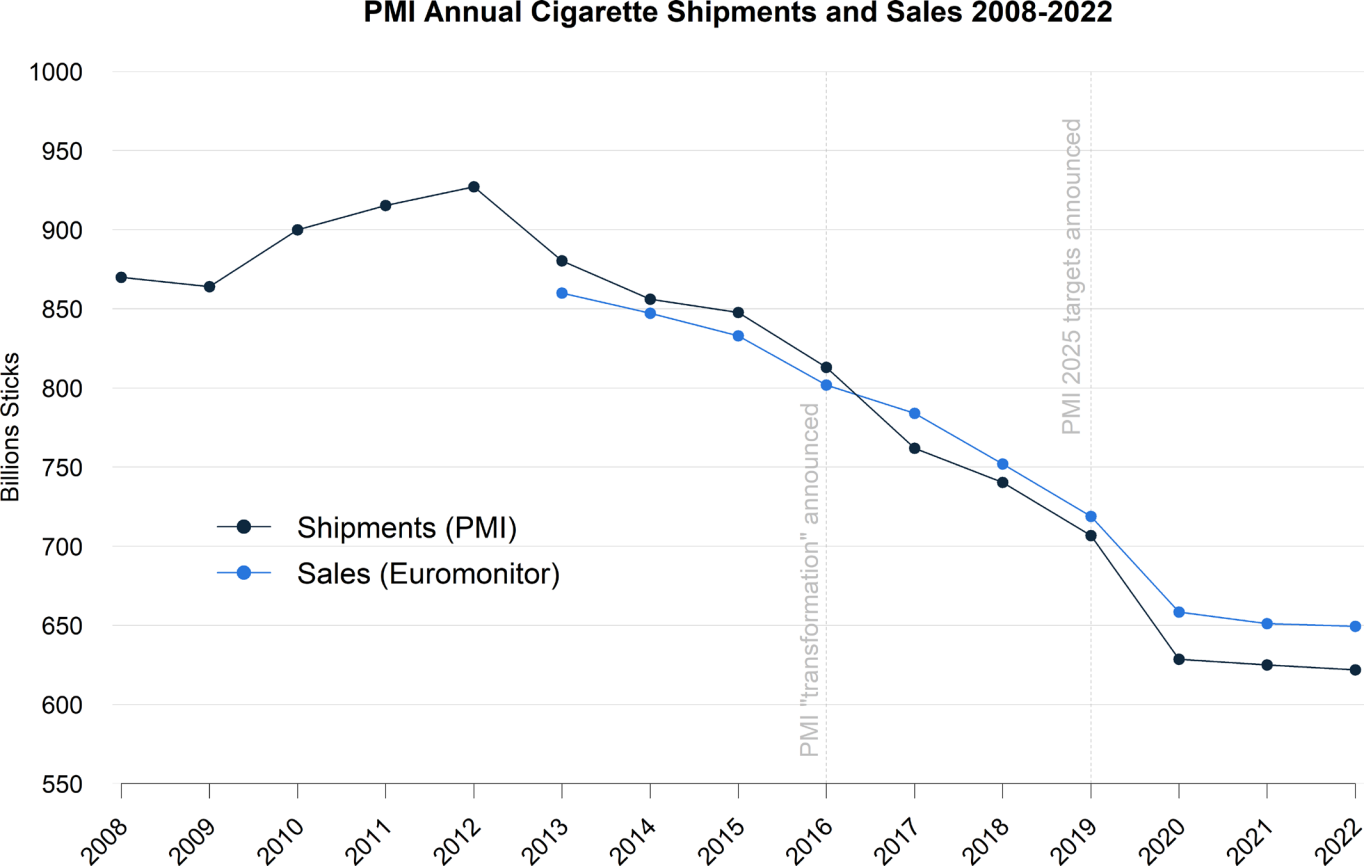
PMI reported annual cigarette shipment volumes compared with PMI annual global sales data extracted from Euromonitor (July 2023). Note that although Euromonitor provides total global and region specific sales data dating back to 2008, it only provides company specific sales data for a period of 10 years before the date of data extraction (from 2013 in this instance). PMI, Philip Morris International.

Figure 1 shows a comparison of PMI-reported cigarette shipment volume data and worldwide PMI cigarette sales data from Euromonitor, clearly showing that the sales and shipments values are similar but not identical. The trends over the period 2013–2020 show shipments falling by approximately 34.3 billion sticks per year, with sales falling at a slightly lower rate, 27.5 billion sticks per year. Finally, in the period 2020–2022, the shipments and sales data exhibit a very similar trend, with respective yearly declines of approximately 3.5 and 4.5 billion sticks, totalling 1% and 1.3% declines over the 2-year period.

Some difference between PMI shipment and sales data is expected, due to existing inventory in the market. However, cumulative values over time tend to align to smooth out this inventory effect: over the period 2013–2020 the Euromonitor total sales differ from the total shipments by only 0.3%. So, even though the trends differ slightly, the overall volumes match more closely.

**Figure 2:**
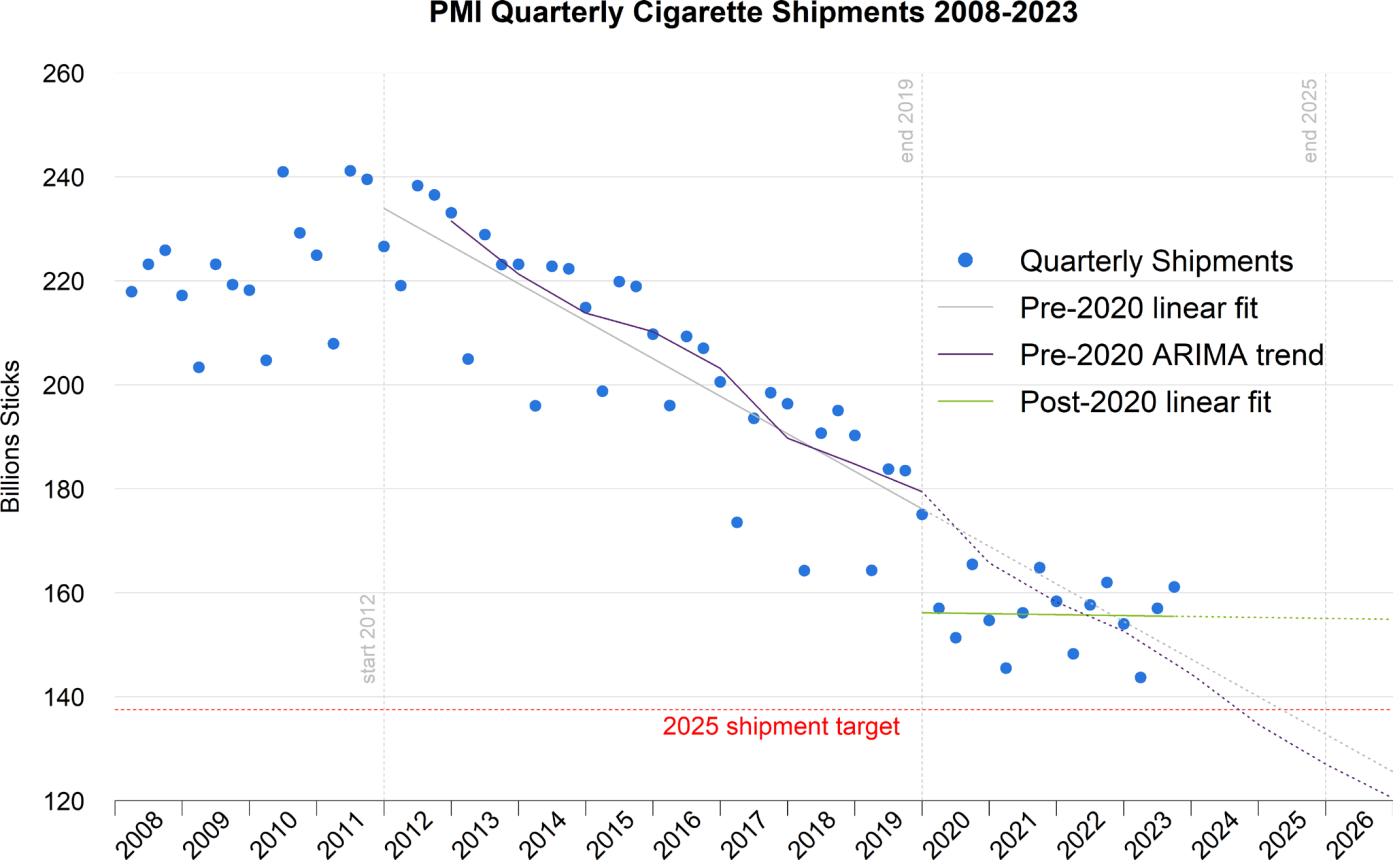
PMI quarterly cigarette shipment volumes (dots), Q1 2008–Q3 2023, with linear regression fits before and after the 2019 Integrated Report, and the average trend from an ARIMA model of the pre-2020 data. The solid lines are the model fits in the relevant fit ranges, and the dotted lines of the same colour are the forecast trends beyond the fit ranges for the different models in each case. PMI’s 2025 cigarette shipment target, expressed as a quarterly volume of 137.5 billion sticks, is also shown. PMI, Philip Morris International.

Figure 2 shows PMI quarterly shipment volumes of cigarettes in the period 2008–2023, with the pre-2020 and post-2020 linear regression fitted and forecast trends shown, as well as the pre-2020 ARIMA fitted and forecast trends. The pre-2020 ETS fitted and forecast trends are not displayed, as they overlap almost entirely with the ARIMA trends, and their inclusion would thus add unnecessary clutter to the graph (see online supplemental table S1 for detailed values). Similarly, the post-2020 ARIMA and ETS trends are completely flat and are barely distinguishable from the post-2020 linear regression fit, so again are not displayed on the graph to maintain clarity (see online supplemental table S2).

Looking at the pre-2020 trends, all three models show that the PMI 2025 shipment target of 550 billion cigarettes would be met by at least 2025 based on this pre-existing trend alone. The linear regression model fits the data quite well, with adjusted R^2^=0.698 and overall p<0.001, and a slope of −7.23 billion cigarettes per quarter. The best-fit ETS model is one with additive trend, seasonality and error while the best-fit ARIMA model is (1,0,0) (2,1,0)_4_. The best fit ARIMA and ETS models both forecast the 2025 target to be met by the end of 2024.

However, the trend in cigarette shipments post-2020 shows that the decline in cigarette shipments has stalled considerably, and in fact, all three models are consistent with a flat or slightly decreasing trend in annual cigarette shipments post-2020. The best-fit ARIMA model is (0,0,0) (0,1,0)_4_, indicating that the data are best modelled as noise in the differences between seasonal values, and similarly the best-fit ETS model is one without seasonal or trend components and just comprises a multiplicative noise term. Likewise, the linear regression model is a poor fit to the data (adjusted R^2^=−0.076 and overall p=0.911), showing there is no statistically significant dependence on time in the linear fit to the data, even if the slope of the regression line shows a marginal decrease of 0.18 billion cigarettes per quarter.

Importantly, given that the slope of the pre-2020 linear fit is statistically significant from a flat line (p<0.001), the change in trend can be said to be significant at this level.

The forecast annual shipment volumes in 2025 from all three models, for both the trends pre-2020 and post-2020, are shown in table 1, with fitted and forecast values for other years given in online supplemental tables S1, S2).

**Table 1:** Point forecasts for annual cigarette shipments (in billions of sticks) at the end of 2025 for each of the three models used in the study and based on the trends before and after the 2020 announcement

	Linear regression	ARIMA	ETS
**Pre-2020 trend**	549.2	508.1	510.3
**Post-2020 trend**	620.6	615.9	623.2

ARIMA, auto regressive integrated moving average; ETS, error, trend, seasonal.

So, in summary, PMI did not have to alter the existing declining trend in its cigarette shipments to meet its announced target, which should easily have been met by 2025 on the basis of the preannouncement trends alone. Moreover, from January 2020, the declining trend halted almost completely, and on the basis of this later trend, PMI’s 2025 target will not be met, staying at a level approximately 12.5%, or 70 billion cigarettes, higher than the target. There is consistent agreement on these fitted and forecast trends across the three modelling approaches taken in the study.

**Figure 3:**
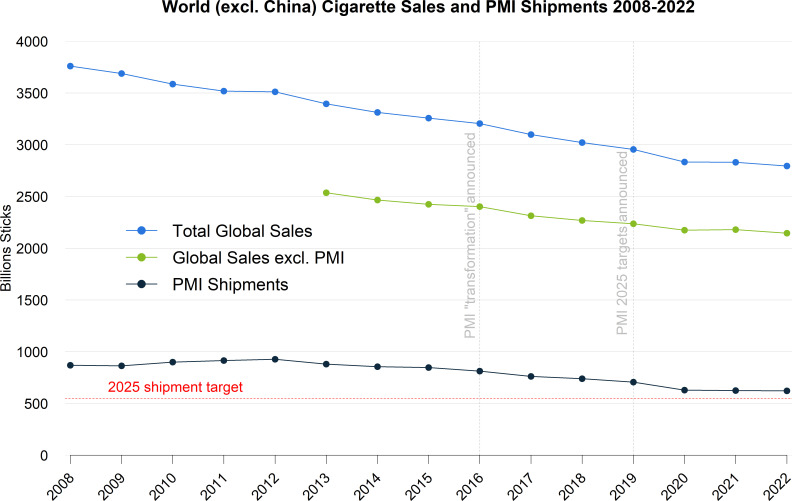
Global (World excluding China) cigarette sales 2008–2022 from Euromonitor (extracted July 2023), with PMI annual shipments for the same period. Global sales excluding PMI sales are also shown for the period for which Euromonitor PMI sales data are available (2013–2020). PMI,Philip Morris International.

To add further context, the trends in total global (world excluding China) cigarette sales, with and without PMI sales, and PMI cigarette shipments are shown in figure 3. China is excluded, as the cigarette market in China, although large in a global context (approximately 47% of all cigarette sales in 2022^11^), is almost entirely dominated by the China National Tobacco Company, and its inclusion is not relevant to the operating environment of transnational tobacco companies such as PMI.^13^

Figure 3 shows that global cigarette sales declined at an approximately consistent rate from 2008 to 2020, whereas PMI cigarette shipments have only been declining since 2012 (also see figure 1). The recent slow-down (post-2020) in cigarette sales declines observed in the PMI data is also observed in global sales data, even when excluding PMI sales, suggesting this slow-down is an industry-wide phenomenon.

Looking in more detail at cigarette sales, table 2 shows the annualised percentage declines in overall global and regional sales since 2013, and in the global and regional sales of PMI and its largest competitor, British American Tobacco (BAT), with data taken from Euromonitor (July 2023).^11^

**Table 2:** Compound annual growth rates of total global and regional cigarette sales and those of PMI and BAT over the periods 2013–2020 inclusive and 2020–2022 inclusive

	Total		PMI		BAT‡	
	2013–**2020**	2020–**2022**	2013–**2020**	2020–**2022**	2013–**2020**	2020–**2022**
World*	−2.56%	−0.69%	−3.73%	−0.84%	−1.77%	−2.98%
**Europe†**	−3.43%	−3.01%	−3.32%	−2.04%	−0.80%	−5.40%
**Americas†**	−3.54%	−3.35%	−4.73%	−1.45%	−3.92%	−3.82%
**Middle East and Africa**	1.54%	1.32%	0.39%	3.00%	−0.84%	−4.49%
Asia-Pacific*	−3.35%	0.83%	−5.08%	−0.35%	−0.63%	1.50%
**Australasia**	−6.72%	−11.6%	−10.9%	−15.7%	−7.35%	−13.7%

* World and Asia-Pacific data excludes China.

† Region definitions are those used by Euromonitor, with Eastern and Western Europe combined as ‘Europe’ and North America and Latin America combined as ‘Americas’.

‡ BAT data pre-2017 include sales figures of Reynolds American, which BAT acquired in full in 2017, allowing a truer comparison of current BAT sales with its historical sales.

BAT, British American Tobacco; PMI, Philip Morris International.

Table 2 shows that over the period 2013–2020, PMI’s cigarette sales declined at a faster rate year-on-year (−3.73%) than the total global market for cigarettes (−2.56%) and faster than BAT’s sales. Consistent with the shipment data analysed above, this decline in total global cigarette sales lessened considerably since 2020. Notably, the yearly rate of decline of PMI’s sales since 2020 (−0.84%) is comparable to the global rate of decline (−0.69%) but much less than that of BAT (−2.98%).

By region, cigarette sales were declining from 2013 to 2020 across all regions except Middle East and Africa. A slight slow-down in decline since 2020 is evident in Europe and the Americas with Asia-Pacific recording an increase in sales. The rate of decline increased in Australasia. At a company level, the regional picture is mixed. Before 2020, PMI’s pattern of changes in sales was similar to the global figures. The decline in PMI sales slowed after 2020 in all regions except Australasia. Sales increased to a greater extent in the Middle East and Africa. BAT sales declined more since 2020 in Europe, Middle-East and Africa, and Australasia, but sales increased in Asia-Pacific during this period.

## Discussion

Our analysis shows that PMI did not set a genuinely transformative or ‘aspirational’ target for reduced cigarette shipments by 2025 but a conservative target which, given the pre-existing trends in its own data, it must have known it could easily meet. It also shows that from 2020 there has been a significant change—a marked stalling in the decline in sales—such that, on post-2020 trends, PMI will not even make that very conservative target. We show that this stalling in the rate of decline in cigarette sales has also been seen globally, raising concerns about progress in global tobacco control during a period of intense industry lobbying^6 14^ and increasing industry interference.^15^ This observed stalling in cigarette sales matches other evidence showing a slowing down of the decline in smoking prevalence globally and across diverse regions of the world.^16 17^

PMI has sought to portray itself as a company ‘transforming’ to a ‘smoke-free’ business^1 8^ and has developed a profitable HTP business, with diversification into other tobacco and nicotine products including through the acquisition of Swedish Match in 2022.^18^ In 2022, PMI reported that its revenues from these non-cigarette activities constituted almost 32% of total revenues, on the back of shipments of 110 billion heated tobacco sticks, almost a doubling of its 2019 shipments.^5^ By comparison, BAT reports its non-cigarette revenue as 15% of the total in 2022,^19^ although it has a larger share in the global e-cigarette market.^11^

However, from a public health perspective, it is the trajectory of the decline in shipments and sales of cigarettes that is of most relevance. One definition of a transforming tobacco company is ‘one demonstrating substantial, rapid and verifiable progress towards eliminating the production and sale of conventional tobacco products within 5 years in all markets where it operates’.^3^ The conservative 2025 target for reducing combustible tobacco product sales, coupled with the significant slowing in decline in sales and shipments since 2020 suggesting PMI is unlikely to make this target, is inconsistent with true transformation. The data presented here show a company that is not so much transforming, as one retaining a sizeable cigarette business (the largest cigarette company by sales outside China in 2022, according to Euromonitor^11^), where the decline in sales has stabilised while introducing a second business arm (HTPs) that is a driver of significant extra revenue.

This finding is consistent with the two sides to its rhetoric observed in analysis of its outputs—a public-facing rhetoric that claims a commitment to public health goals and an investor-facing rhetoric that focuses on business as usual (maintaining revenue and profits).^9^ It provides further evidence that PMI is not transforming but is instead seeking to harness genuine public health interest in harm reduction to secure influence and undermine progress in tobacco control,^14 20 21^ a strategy it has a history of using.^22^,^24^

### Strengths and limitations

A major strength of the study is the use of three different methods for modelling the shipment data (linear regression, ARIMA and ETS), with the forecast results from all three methods broadly agreeing, giving confidence in the results presented.

It is noted that the flattening in PMI’s cigarette shipments and sales post-2020 is also observed in overall global and regional cigarette sales data. This trend is highly worrying from a public health standpoint. However, it is based on only three data points and ongoing monitoring of trends will be important to determine if this is a sustained trend. Relatedly, only descriptive statistics of regional and other tobacco company sales are presented here, and a more robust statistical analysis of these trends is warranted when data from further time points becomes available enabling this. Furthermore, a broader analysis by region, market and company, not just of cigarette sales but also of ‘non-combustible’ tobacco products would be useful future research.

Finally, the broad agreement between PMI’s own reported shipment trends and sales trends reported by Euromonitor provides confidence in the robustness of the results. Euromonitor is a widely used and trusted source for market data, but it is also important to recognise that Euromonitor has received funds from PMI, starting in 2019,^25^ and a consideration for future studies on trends of cigarette sales would be to also include other sources of sales data, for example, GlobalData.^26^

## Conclusion

PMI’s 2025 target for cigarette shipments is not an ambitious, transformative change for the company but simply a reflection of existing trends in cigarette sales at the time the target was announced; trends which reflect the success of global tobacco control efforts to that point. Moreover, since the announcement, the declining trend in PMI’s cigarette shipments has flattened considerably, making it very unlikely that it will even meet its own, very conservative target. With a projected level in 2025 of an extra 70 billion cigarette shipments above an already inadequate target, this does not support its narrative of transforming into a ‘smoke-free’ company.

## Supplementary material

10.1136/tc-2023-058511 online supplemental file 1

## Data Availability

Data are available on reasonable request. Data may be obtained from a third party and are not publicly available.
