# Supplementary material for: Analysis of Philip Morris International’s ‘aspirational’ target for its 2025 cigarette shipments
Source: Tob Control. 2024 May 23;34(e1):e058511. doi: 10.1136/tc-2023-058511 (PMC12772595; doi:10.1136/tc-2023-058511)
Supplement: online supplemental file 1 [file tc-34-e1-s001.pdf]

Supplementary Tables

Supplementary Table S1: Yearly total cigarette shipment volumes (billions of sticks) 2012-2026 as fitted and forecast by three different models to the quarterly shipment data 2012-2019. Actual yearly shipments are also included for reference.

Year	Actual		Linear Regression	ARIMA	ETS
**2012**	927.0	**Fitted**	924.9	926.1	928.3
**2013**	880.2		896.0	885.1	886.4
**2014**	856.0		867.1	855.3	852.4
**2015**	847.3		838.2	841.0	838.4
**2016**	812.9		809.3	812.6	815.3
**2017**	761.9		780.4	758.9	763.1
**2018**	740.3		751.5	739.1	734.6
**2019**	706.7		722.6	717.7	713.6
**2020**	628.5	**Forecast**	693.7	663.1	671.3
**2021**	624.9		664.8	632.9	639.1
**2022**	621.9		635.9	610.5	606.9
**2023**			607.0	577.6	574.7
**2024**			578.1	538.7	542.5
**2025**			549.2	508.1	510.3
**2026**			520.3	482.1	478.1

Supplementary Table S2: Yearly total cigarette shipment values (billions of sticks) 2020-2026 as fitted and forecast by three different models to the quarterly shipment data 2020-2023. Actual yearly shipments are also included for reference. *Yearly value for 2023 is combination of fitted values for the first three quarters and forecast value for the last quarter.

**Year**	**Actual**		**Linear Regression**	**ARIMA**	**ETS**
**2020**	628.5	**Fitted**	624.2	627.9	623.2
**2021**	624.9		623.5	628.5	623.2
**2022**	621.9		622.8	624.9	623.2
**2023**		**Forecast***	622.1	621.9	623.2
**2024**			621.0	615.9	623.2
**2025**			620.6	615.9	623.2
**2026**			619.9	615.9	623.2
